# Mutation and prognostic analyses of PIK3CA in patients with completely resected lung adenocarcinoma

**DOI:** 10.1002/cam4.852

**Published:** 2016-08-23

**Authors:** Zhengbo Song, Xinmin Yu, Yiping Zhang

**Affiliations:** ^1^ Department of Medical OncologyZhejiang Cancer HospitalHangzhou310022China; ^2^ Key Laboratory Diagnosis and Treatment Technology on Thoracic OncologyHangzhouZhejiang310022China

**Keywords:** Frequency, non‐small‐cell lung cancer, overall survival, PIK3CA mutation, treatment

## Abstract

PIK3CA mutation represents a clinical subset of diverse carcinomas. We explored the status of PIK3CA mutation and evaluated its genetic variability, treatment, and prognosis in patients with lung adenocarcinoma. A total of 810 patients with completely resected lung adenocarcinoma were recruited between 2008 and 2013. The status of PIK3CA mutation and other three genes, that is, EGFR mutation, KRAS mutation and ALK fusion were examined by reverse transcription‐polymerase chain reaction (RT‐PCR). Survival curves were plotted with the Kaplan–Meier method and log‐rank for comparison. Cox proportional hazard model was performed for multivariate analysis. Among the 810 patients, 23 cases of PIK3CA mutation were identified with a frequency of 2.8%. There were 14 men and 9 women with a median age of 61 years. Seventeen tumors revealed concurrent gene abnormalities of EGFR mutation (*n* = 12), KRAS mutation (*n* = 3), and ALK fusion (*n* = 2). Seven patients with EGFR & PIK3CA mutations recurred and administrated of EGFR‐TKIs yielded a median progression free‐survival of 6.0 months. Among four eviromous‐treated patients, stable disease was observed in three patients with a median Progression‐free survival (PFS) of 3.5 months. Patients with and without PIK3CA mutation had different overall survivals (32.2 vs. 49.6 months, *P *=* *0.003). Multivariate analysis revealed that PIK3CA mutation was an independent predictor of poor overall survival (HR = 2.37, *P *=* *0.017). The frequency of PIK3CA mutation was around 2.8% in the Chinese patients of lung adenocarcinoma. PIK3CA mutation was associated with reduced PFS of EGFR‐TKIs treatment and shorter overall survival.

## Introduction

Lung cancer is currently a leading cause of cancer‐related mortality in China 1. Non‐small‐cell lung cancer (NSCLC) accounts for around 80% of lung cancers. Most NSCLC patients have already reached an advanced stage at diagnosis; so, palliative chemotherapy becomes a major option 2. However, the efficacy has been rather disappointing 3. Molecular therapy has become a newly emerging regimen over the last decade 4. With lesser toxicity, patients with sensitive molecular alterations could benefit more from an inhibitor therapy than traditional chemotherapy 5, 6, 7, 8, 9, 10.

PIK3CA gene is known to encode p110*α*catalytic subunit of PI3K protein. Its mutation leads to constitutive activation of protein kinase B signaling, which plays an important role in various physiological and pathological cellular processes 11. PIK3CA mutation was detected in a large variety of human cancers. With a frequency of 2–7% in NSCLC, it is more frequent in lung squamous cell carcinoma than in lung adenocacinoma 12, 13, 14. PIK3CA mutation was found both in patients without EGFR‐TKIs dosing and those resistance to targeted therapy 14, 15. However, because of heterogeneity and insufficient data of previous studies, no conclusion was observed for the frequency and prognosis of lung adenocarcinoma patients with PIK3CA mutation. In addition, it is not well investigated that the option treatment for patients who harbored PIK3CA mutation, especially for patients with PIK3CA and EGFR concurrent gene alterations.

In this study, PIK3CA mutation was screened from 810 lung adenocarcinoma patients and its frequency, treatment and prognosis was evaluated, in order to enrich the understanding of PIK3CA as a driver gene in NSCLC treatment.

## Materials and Methods

### Patient eligibility

Between January 2008 and October 2013, the patients of lung adenocarcinoma undergoing complete resection at our hospital were selected. Histological typing was assessed according to the 2004 pathology classification scheme of World Health Organization (WHO). The seventh TNM classification was adopted for tumor staging. Written informed consent was obtained for gene analysis and the study protocol approved by our institutional Ethics Committee.

### Gene detection

Genomic DNA and RNA were extracted from tumor tissues according to the standard protocols (RNeasy Mini Kit, and QiAamp DNA Mini Kit, Qiagen, Hilden, Germany). Briefly isolated RNA samples were used for reverse transcription into cDNA using Revert Aid First Strand cDNA Synthesis Kit (Fermentas, St Leon‐Rot, Germany). Either genomic DNA or cDNA was employed for polymerase chain reaction (PCR) amplification and sequencing. And EGFR, KRAS, and PIK3CA were amplified by PCR using genomic DNA. Cycle sequencing of purified PCR products was conducted with PCR primers using ADx Mutation Detection Kit (Amory, Xiamen, China). ALK was detected by PCR with Fusion Gene Detection Kit (Amory). The handling procedures were detailed previously 16.

### Follow‐ups and statistical analyses

The follow‐ups were conducted every 3–6 months after chemotherapy and/or radiotherapy. And the last follow‐up date was November 30, 2015.

Categorical variables were compared by chi‐squared test. Kaplan–Meier method was employed for survival analysis and log‐rank for comparison between different groups. Overall survival (OS) was defined as the time from the start of confirmed pathology to the date of death or the last follow‐up. Recurrence‐free survival (RFS) refers to the time of surgery to recurrence or the last follow‐up. Progression‐free survival (PFS) encompassed the time from the therapy to documented progression or death from any cause. Univariate and multivariate analyses were performed with a Cox proportional hazard model. Statistical analysis was conducted with SPSS 18 software (SPSS Inc., Chicago, IL).

## Results

### Patients' profile

A total of 23 patients with PIK3CA mutation were recruited. There were 14 men and 9 women with a median age of 61 years. The smokers were categorized into ever/current (*n* = 10) and never (*n* = 13). The pathologic stages at diagnosis were I (*n* = 9), II (*n* = 4), and IIIA (*n* = 10). Based upon the adenocarcinoma classification scheme, the predominant subtypes were solid (*n* = 8), acinar (*n* = 4), papillary (*n* = 3), micropapillary (*n* = 6), and lepidic (*n* = 2), respectively. Comparisons of PIK3CA mutation‐positive and ‐negative patients are detailed in Table 1.

**Table 1:** Comparison of clinical characteristics between patients with and without PIK3CA mutation

	PIK3CA positive (*n* = 23) (%)	PIK3CA negative (*n* = 787) (%)	*P*
Gender			0.48
Male	14 (60.9)	421 (53.5)	
Female	9 (39.1)	366 (46.5)	
Age			0.35
<60 year	10 (43.5)	415 (52.7)	
≥60 year	13 (56.5)	362 (47.3)	
Smoking status			0.92
Never	13 (56.5)	437 (55.5)	
Former/current	10 (43.5)	350 (44.5)	
Stage at diagnosis			0.74
I–II	13 (56.5)	472 (60.0)	
IIIA	10 (43.5)	315 (40.0)	
Surgical procedures			0.41
Lobectomy	19 (82.6)	689 (87.5)	
Pneumonectomy	1 (4.3)	45 (5.7)	
Others	3 (13.1)	53 (6.8)	
Adjuvant treatment			0.99
Yes	18 (78.3)	615 (78.1)	
No	5 (21.7)	172 (21.9)	

### Genetic analysis

The genetic profiles were EGFR mutation (*n* = 401, 49.5%), KRAS mutation (*n* = 65, 8.0%), ALK arrangement (*n* = 47, 5.8%), and PIK3CA mutation (*n* = 23, 2.8%). The loci of PIK3CA mutation were at exon 9 (*n* = 14) and exon 20 (*n* = 9). Fourteen of 23 patients were of E542K and E545K subtypes. Seventeen tumors had concurrent genetic abnormalities of EGFR mutation (*n* = 12), KRAS mutation (*n* = 3), and ALK fusion (*n* = 2). The details are summarized in Table 2.

**Table 2:** Clinical characteristics of patients with PIK3CA mutation (*n* = 23)

Case	Gender/age	Stage	Concurrent genes	EGFR‐TKI	Everolimus	PFS of EGFR‐TKI	PFS of everolimus	OS
1	M/69	IA	EGFR	–	–	–	–	62.3M
2	M/43	IIIA	KRAS	–	–	–	–	53.7M
3	F/51	IIIA	EGFR	Icotinib	–	7.0M		44.3M
4	M/74	IB	EGFR		–			41.1M
5	F/60	IA	ALK	–	–	–	–	33.1M+
6	M/64	IB	KRAS		–			48.9M+
7	M/45	IIIA	–	Gefitinib	Yes	1.5M	3.0M	12.5M
8	M/62	IIA	EGFR	Icotinib	–	11.0M		24.7M+
9	F/75	IIIA	KRAS	–	–	–	–	21M
10	F/69	IB	EGFR	–	–			19M+
11	M/69	IIB	EGFR	–	–	–	–	32.2M
12	M/61	IIIA	EGFR	Icotinib	–	4.0M		12.0M
13	M/55	IIIA	ALK		–			21M
14	F/68	IB	–	Icotinib	Yes	2.5M	4.0M	26M
15	M/64	IB	–		–			16.8M+
16	M/63	IB	–	–	–	–	–	16M+
17	M/61	IIB	EGFR	Gefitinib	Yes	5.5M	1.5M	17.8M
18	F/31	IIIA	EGFR	Gefitinib	–	8.5M		21M
19	F/43	IIIA	EGFR	Erlotinib	–	–	–	17M
20	M/59	IIIA	–		–	1.2M		17M
21	F/61	IIIA	–	–	–			16M+
22	F/44	IIA	EGFR	Icotinib	Yes (continue icotinib)	9.5M	6.0M	32M
23	M/58	IB	EGFR		–			24M+

M: male; F: female; PFS: progression‐free survival; M: month; OS: overall survival.

### Treatment

Fifteen patients with PIK3CA mutation recurred after resection, including single PIK3CA mutation (*n* = 4) and concurrent gene abnormalities (*n* = 11). Among them, nine received EGFR‐TKIs (EGFR concurrent gene, *n* = 7; single PIK3CA mutation, *n* = 2).

**Figure 1:**
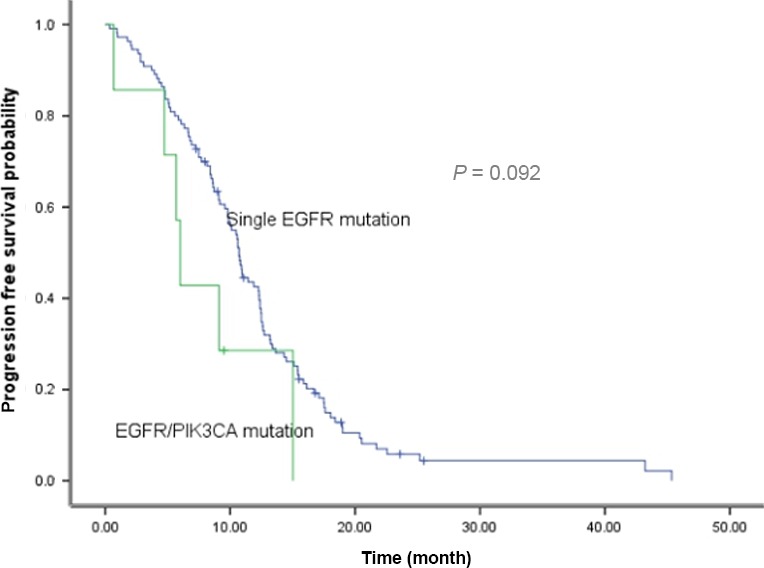
Comparison of progression‐free survival with EGFR‐TKI treatment between patients with single EGFR mutation and EGFR/PIK3CA concurrent alterations.

Among 401 patients with EGFR mutation, 121 received EGFR‐TKIs after recurrence (single EGFR mutation, *n* = 110). There was a general trend of longer PFS for patients with single EGFR mutation than those with concurrent EGFR/PIK3CA mutations (10.7 vs. 6.0 months, *P *=* *0.092) (Fig. 1). Four patients, including single PIK3CA mutation (*n* = 2) and EGFR concurrent mutation (*n* = 2), received everolimus (a mTOR inhibitor). There were stable disease (*n* = 3) and progression disease (*n* = 1). The median PFS was 3.5 months. Notably, one patient with EGFR concurrent mutation progressed from prior gefitinib therapy to gefitinib and everolimus with a 6‐month PFS.

### Survival

**Figure 2:**
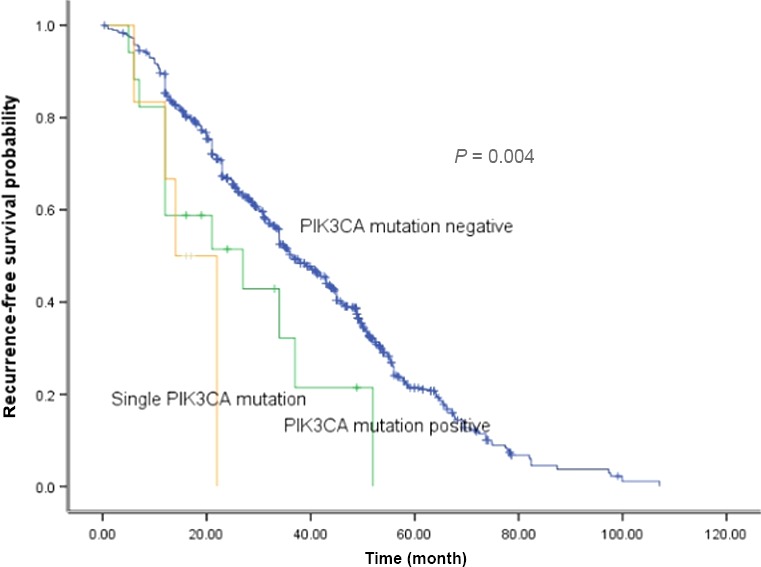
Comparison of recurrence‐free survival among different groups.

The clinical outcomes were recurrence (*n* = 502) and death (*n* = 415). The median RFS and OS of all the 810 patients were 38.0 (95% CI: 34.9–41.0) and 49.1 months (95% CI: 47.2–51.0). The 5 years of RFS and OS rates were 23.5% and 31.3%, respectively. They were divided into three groups—PIK3CA wild‐type (*n* = 787), PIK3CA concurrent (*n* = 17), and single PIK3CA mutation (*n* = 6). The median RFS for three groups were 36.5, 27.0, and 14.0 months, respectively (*P *=* *0.004) (Fig. 2).

**Figure 3:**
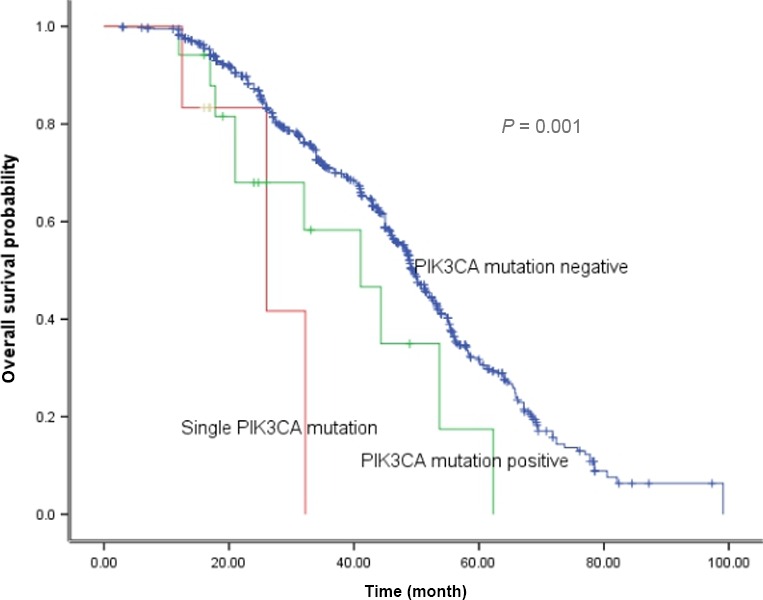
Comparison of overall survival among different groups.

**Figure 4:**
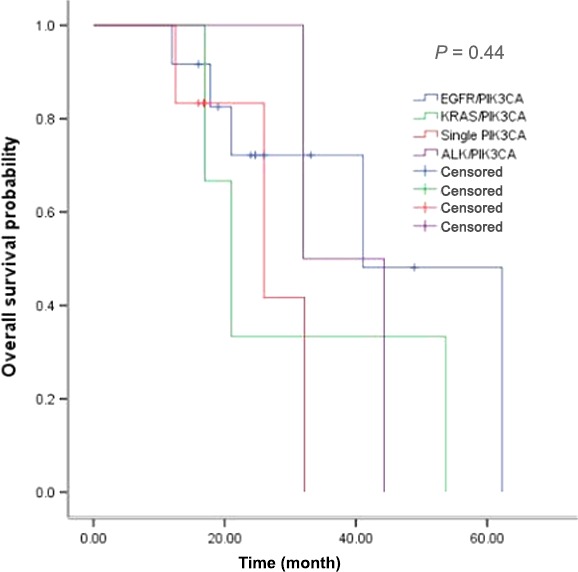
Comparison of overall survival among single PIK3CA mutation, EGFR/PIK3CA mutation, KRAS/PIK3CA mutation, and ALK/PIK3CA mutation.

The median OS for PIK3CA wild‐type, PIK3CA concurrent mutation, and single PIK3CA mutation were 49.6, 41.1, and 26.0 months, respectively (*P *=* *0.001) (Fig. 3). No survival difference existed among single PIK3CA mutation, EGFR/PIK3CA mutation, KRAS/PIK3CA mutation, and ALK/PIK3CA mutation patients (*P *=* *0.44) (Fig. 4).

The results of univariate and multivariate Cox's regression analyses are summarized in Table 3. Late‐stage (stage III) and PIK3CA mutation were correlated significantly with worse OS. No significant differences in OS existed among age, gender, adjuvant treatment, or smoking status. Multivariate analysis revealed that PIK3CA mutation and late stage were two independent prognostic factors for worse survival.

**Table 3:** Univariate and multivariate Cox's regression analysis of overall survival

Variables	Univariate analysis			Multivariate analysis		
	HR	95% CI	*P*	HR	95% CI	*P*
Age (<60 vs. ≥60 years)	1.06	0.86–1.31	0.57	–	–	–
Gender (female vs. male)	0.95	0.77–1.16	0.59			–
Stage (III vs. I + II)	1.53	1.36–1.71	0.00	1.51	1.35–1.70	0.00
Smoking status (nonsmokers vs. smokers)	0.97	0.83–1.14	0.71	–	–	–
Adjuvant treatment (Yes vs. no)	0.77	0.49–1.23	0.28	–	–	–
PIK3CA mutation (Yes vs. no)	2.26	1.30–3.95	0.004	2.37	1.17–4.83	0.017

HR: hazard ratio, CI: confidence interval.

## Discussion

In present study, we found that PIK3CA mutation occurred in approximately 2.8% of patients with lung adenocarcinoma in a cohort of Chinese patients. Furthermore, we detected that it was commonly concurrent with EGFR mutation and the patients with EGFR/PIK3CA had a shorter PFS than those with single EGFR mutation. It also found that everolimus might be selected for patients with PIK3CA mutation. In addition, PIK3CA mutation was identified as a prognostic factor for poor RFS and shorter OS. To the best of our knowledge, this study represents the first‐ever attempt of focusing on PIK3CA mutation for lung adenocarcinoma and detecting the genetic variability, treatment, and prognosis.

With a frequency range of 2–5%, PIK3CA mutation occurred more in squamous cell carcinoma than lung adenocarcinoma. The incidence of PIK3CA mutation was around 3% in lung adenocarcinoma and 5–10% in squamous cell carcinoma 12, 13, 14, 15. In this study, the frequency of PIK3CA mutation at 2.8% was consistent with that of previous studies.

Although single PIK3CA mutations have been reported 17, the majority of lung cancer with PIK3CA mutations had another cocurrent driver mutation in previous studies. Sixteen of twenty‐three patients harboring PIK3CA mutations were found to be with coexisting driver genes in one study by Chaft et al., and with KRAS mutation most concurrently 18. Different from Chaft et al. study based on Caucasian populations, EGFR mutation was identified as a dominant gene in Asian population, which may be a determined factor for EGFR concurrent with other genes. In this study, 17 of 23 patients had concurrent genes and PIK3CA/EGFR co‐current was predominant. Considering previous studies and ours, we concluded that PIK3CA mutation commonly coexisted with other genes, although with a rare frequency.

It is widely identified that patients of NSCLC harboring EGFR mutation could benefit from EGFR‐TKI treatment and the median PFS ranged from 10–13 months 2, 3, 4. However, more than 10% of the patients with EGFR mutation did not respond to EGFR‐TKIs treatment. Concurrent gene with EGFR mutation may affect the efficacy of EGFR‐TKIs based on previous studies. The PIK3CA pathways play an important role in various physiological and pathological processes, such as proliferation, differentiation, and apoptosis. EGFR activation elicits its effects partially via PIK3CA/AKT/mTOR pathways, which promote tumor proliferation, invasion, and migration. Mutation in PIK3CA signaling pathways may induce resistance to EGFR‐TKIs of lung cancer patients. Although reported in previous studies, treatment and efficacy data of patients harboring PIK3CA mutations were not available 12, 13. The results of a meta‐analysis showed that PIK3CA mutation adversely affected the clinical efficacy and OS of NSCLC patients on EGFR‐TKIs 14. However, only 12 patients from four studies were enrolled in this meta‐analysis. In contrast, concurrent PIK3CA and EGFR mutations did not compromise the clinical benefits of EGFR‐TKI dosing in another study by Eng, et al 19. Similarly, there were only 10 patients with PIK3CA mutation in Eng, et al. Study. Seven cases with concurrent PIK3CA/EGFR mutations received EGFR‐TKIs and yielded a median PFS of 6.0 months in current study and the PFS was slightly shorter than that of single EGFR mutation. Together previous and our study, we can concluded that PIK3CA may influence the clinical efficacy of EGFR‐TKIs based on previous and present study. Clinically, everolimus, a mTOR inhibitor, has been widely prescribed for advanced renal carcinoma and breast carcinoma 20, 21. A moderate efficacy of everolimus was demonstrated for NSCLC in previous studies 22, 23. A combined use of EGFR‐TKIs and everolimus was found to be more effective than everolimus alone in one study 22. Four patients had a median PFS of 3.5 months in this study. One patient who progressed from prior EGFR‐TKI received EGFR‐TKI and everolimus and achieved a 6‐month remission. However, it has been unclear whether or not PIK3CA mutation is a clinical marker for everolimus treatment. From the efficacy analysis of current study with limited number of patients, we concluded that PIK3CA mutation may play some role in affecting the EGFR‐TKI efficacy and is a potential indicator of mTOR inhibitor treatment in NSCLC, but needs to be validated with a large number of patients. From the perspective of current study, PIK3CA mutations should be detected as a routine practice simultaneous with EGFR mutations.

Because of the rarity of PIK3CA mutation, its prognostic value with regard to carcinomas has not been thoroughly examined in previous studies 14. Existing data have remained inconclusive. Although better prognosis was reported in breast cancer with PIK3CA mutation, it was an unfavorable prognostic factor for colorectal cancer and NSCLC 14, 24, 25. The coexistence of PIK3CA and other driver genes may affect the analysis of PIK3CA as a prognostic marker. Our study revealed that the patients with single PIK3CA mutation had the shortest OS. It suggested that PIK3CA mutation may be a predicator of poor prognosis for lung adenocarcinoma.

Some inherent limitations of our study lay in its retrospective design and a small number of patients with PIK3CA mutation. Only 15 of 23 patients harboring PIK3CA mutation had recurrence, which may affected the clinical efficacy analysis of PIK3CA mutation as predict and prognosis factor in lung adenocarcinoma.

In summary, PIK3CA mutation represents a subset of lung carcinoma with a prevalence of 2.8% in Chinese population. The status of PIK3CA mutation may impact the clinical efficacy of EGFR‐TKIs and PIK3CA mutation may serve as a prognostic factor of poor survival.

## Conflict of Interest

The authors declare no conflicts of interest.
